# The effect of different service models on quality of care in the assessment of autism spectrum disorder in children: study protocol for a multi-centre randomised controlled trial

**DOI:** 10.1186/s12887-022-03244-y

**Published:** 2022-04-02

**Authors:** Thuy T. Frakking, John Waugh, Christopher Carty, Alison Burmeister, Annabelle Marozza, Sue Hobbins, Michelle Kilah, Michael David, Lisa Kane, Susan McCormick, Hannah E. Carter

**Affiliations:** 1 Research Development Unit, Caboolture Hospital, Queensland Health, McKean St, Caboolture, QLD 4510 Australia; 2 Centre for Clinical Research, School of Medicine, The University of Queensland, Herston, QLD 4029 Australia; 3 Speech Pathology Department, Gold Coast University Hospital, Southport, QLD 4215 Australia; 4 School of Clinical Medicine, The University of Queensland, St Lucia, QLD 4067 Australia; 5 Department of Paediatrics, Caboolture Hospital, Queensland Health, McKean St, Caboolture, QLD 4510 Australia; 6 Centre for Musculoskeletal Research, Griffith Health Institute and School of Allied Health Sciences, Griffith University, Gold Coast, 4222 Australia; 7 Speech Pathology Department, The Prince Charles Hospital, Chermside, QLD 4032 Australia; 8 Occupational Therapy Department, Caboolture Hospital, Queensland Health, McKean St, Caboolture, QLD 4510 Australia; 9 Department of Paediatrics, The Prince Charles Hospital, Chermside, QLD 4032 Australia; 10 Nursing Outpatients, Caboolture Hospital, Queensland Health, McKean St, Caboolture, QLD 4510 Australia; 11 School of Medicine, Griffith University, Gold Coast Campus, Gold Coast, QLD 4222 Australia; 12 Australian Centre for Health Services Innovation and Centre for Healthcare Transformation, Queensland University of Technology, Kelvin Grove, Brisbane, QLD 4059 Australia

**Keywords:** Autism spectrum disorder, Paediatrics, Randomised controlled trial, Models of care, Value healthcare

## Abstract

**Background:**

There is significant variability in clinical pathways available in the diagnostic assessment of ASD, including the order and timing of allied health assessments in relation to paediatrician consultations. Allied health professionals in first-contact models are increasingly used to improve the timeliness of healthcare access, whilst complementing medical specialty workforce shortages. Anecdotally, the implementation of allied health first-contact models in paediatrics has improved waitlists and timely access to healthcare. However, no rigorous studies have been conducted to evaluate the outcomes of these models. This study aims to determine the impacts of an allied health first-contact model on health service use and costs and patient quality of life and satisfaction.

**Methods:**

An open, semi-blinded, multi-centre randomised controlled trial in paediatric outpatient clinics at two Australian metropolitan public hospitals. 56 children (0–16 years) fulfilling the inclusion criteria will be randomised to one of two clinical pathways for assessment of ASD: (1) allied health first-contact or (2) medical first-contact model. Cost outcomes will be collected from both health service and family perspectives. Caregiver-reported outcome measures include: Pediatric Quality of Life Inventory (PedsQL), the EuroQOL Five Dimension Youth Version (EQ-5D-Y), the Autism Family Experience Questionnaire (AFEQ) and Measure of Processes of Care.

**Discussion:**

Evidence of improvements in service and consumer centric outcomes will help inform the development and implementation of high-value, evidenced based models of care for the assessment of ASD in children. The findings from this study are expected to contribute to the evidence base around the costs and consequences of allied health first contact models for the assessment of children with ASD in the Australian setting. Findings of this study may help to inform the allocation of health care resources while maintaining, or potentially improving, patient and family quality of life and experience of care. These findings may be useful in informing the wider adoption of these models in Australia and internationally, particularly in healthcare settings where medical specialist shortages exist.

**Trial registration:**

Australia and New Zealand Clinical Trials Register (ANZCTR) ACTRN12621001433897. Registered: 25^th^ October, 2021.

## Background

Autism Spectrum Disorder (ASD) is a neurodevelopmental disorder which is characterized by social-communication impairment and by repetitive patterns of behavior [1]. In Australia, the prevalence of ASD in children is estimated between 2.4% to 3.9% [2]. Early diagnosis of ASD is considered best practice as early intervention for emotional regulation, communication, cognitive, behaviours, physical, sensory processing and/or social skills support provides children with improved educational outcomes and quality of life [3, 4]. The Australian Guidelines for ASD assessment acknowledge the importance of allied health (e.g. psychologist, speech pathologist, occupational therapist) contributions to the clinical diagnosis of ASD within a multidisciplinary team setting [5]. However, in clinical practice there is significant variability in clinical pathways available in the diagnostic assessment of ASD, including the order and timing of allied health assessments in relation to paediatrician consultations.

Allied health professionals in first-contact models are increasingly used to improve the timeliness of healthcare access, whilst complementing medical specialty workforce shortages [6, 7]. Dietitian and Speech Pathology/Audiology first-contact models have been effective in reducing medical specialty waitlists in adult populations [8–10]. A recent Australian study demonstrated that dietitian first-contact models in the management of low-risk gastroenterology conditions resulted in high consumer satisfaction with services and improved quality of life outcomes [8, 9]. This is important in the sustainable delivery of patient-centric, high value healthcare [11].

Within the paediatric population in Queensland, Australia, multidisciplinary allied health first-contact models of care for the assessment of ASD exist across several healthcare facilities [12]. In this model, children are first seen by a psychologist, speech pathologist, occupational therapist and/or social worker before initial contact with a medical specialist (e.g. Paediatrician). Anecdotally, the implementation of allied health first-contact models in paediatrics has improved waitlists and timely access to healthcare. However, no rigorous studies have been conducted to evaluate the outcomes of these models, when compared with traditional medical first-contact models of care. This is a critical gap in the literature as service and consumer centric outcomes are needed to inform the development and implementation of high value, evidence based models of care in the diagnostic assessment of ASD in children. In this study, we will conduct a randomised controlled trial to determine the impacts of an allied health first-contact model on health service use and costs and patient quality of life and satisfaction.

## Methods

### Aims

This aim of this study is to determine if an allied health first-contact model of care reduces health service use and costs without compromising patient quality of life or satisfaction when compared to traditional medical first-contact models of care in the assessment of ASD in children. We hypothesize that an allied health first-contact model of care reduces healthcare use and costs; and results in comparable patient quality of life and satisfaction with care, when compared to a traditional medical first-contact model of care.

### Study design

This trial is designed as an open, semi-blinded, randomised controlled trial within paediatric outpatient clinics located at secondary Australian public hospitals: Caboolture Hospital and The Prince Charles Hospital (TPCH).

### Participants

Children who have been referred to a Paediatrician for assessment of suspected ASD will be eligible to participate in the study. Additionally, enrolled children will meet the following inclusion criteria: aged between 0 to 16 years; referral for a cognitive, behavioural, learning and/or developmental assessment and determination by the triaging team that the assessment of ASD is warranted as part of the differential diagnosis process. Children will be excluded from participation in the study if they have received two or more allied health assessments completed in past 12 months, children in out of home care, a previous diagnosis of ASD and/or acute functional deterioration requiring urgent medical review.

### Recruitment

All referrals will be screened for eligibility by a member of the research team. Informed consent will be obtained and caregivers will be provided with a caregiver information sheet and study brochure. The researcher will verbally go through the caregiver information sheet, study brochure and consent form for all caregivers of eligible participants. Interpreter services may be utilized to assist with obtaining informed consent when caregivers are identified with non-English speaking backgrounds.

It is possible that some caregivers will identify as having literacy issues. In such instances, additional time will be allocated to explain the study and obtain informed consent [13]. The additional time used to obtain informed consent will include the “teach back” method to ensure that the caregivers understand the risks and processes involved in participating in the study [14].

### Randomisation

An independent biostatistician will generate a randomisation list, which will include block size permutations of *n* = 5 to ensure an equal number of participants enrolled in each group and to minimise selection and accidental bias [15]. Model of care allocation (allied health versus medical first pathway) will be assigned by a clinic nurse independent of the research team following enrolment. The opening of concealed sequentially numbered opaque envelopes which contain the group allocation will only be completed by the clinic nurses at each site.

### Blinding

Blinding will be maintained by emphasising to intervention staff and participants that each model of care adheres to best practice principles, and each is advocated by certain professionals to be superior for the assessment of ASD in children. Except for the interventionists (i.e. paediatricians and allied health staff) and clinic nurse, study investigators and staff will be blinded to group allocation of the participants. The trial will adhere to established procedures to maintain separation between the study research assistant that takes outcome measurements and the healthcare staff that deliver the intervention. The research assistant will obtain all outcome measurements and will not be informed of the group assignment. All investigators, staff, and participants will be kept masked to outcome measurements and trial results until the conclusion of the study.

### Outcome measures

Cost outcomes will be estimated from both health service and family perspectives. Health service use and costs will include all allied health and specialist outpatient appointment costs, as well as the length of stay and cost of any hospital admissions. Family costs will include costs associated with travel to appointments, including parking costs, and time off work to attend appointments.

Caregiver-reported outcome measures will be collected using the following validated questionnaires: Pediatric Quality of Life Inventory (PedsQL) [16] child module (Score 0 to 100; parent or child completed), the EuroQOL Five Dimension Youth Version (EQ-5D-Y) [17], the Autism Family Experience Questionnaire (AFEQ) [18] and Measure of Processes of Care [19]. Patient wait times to access care will also be recorded.

### Data collection

**Fig. 1:**
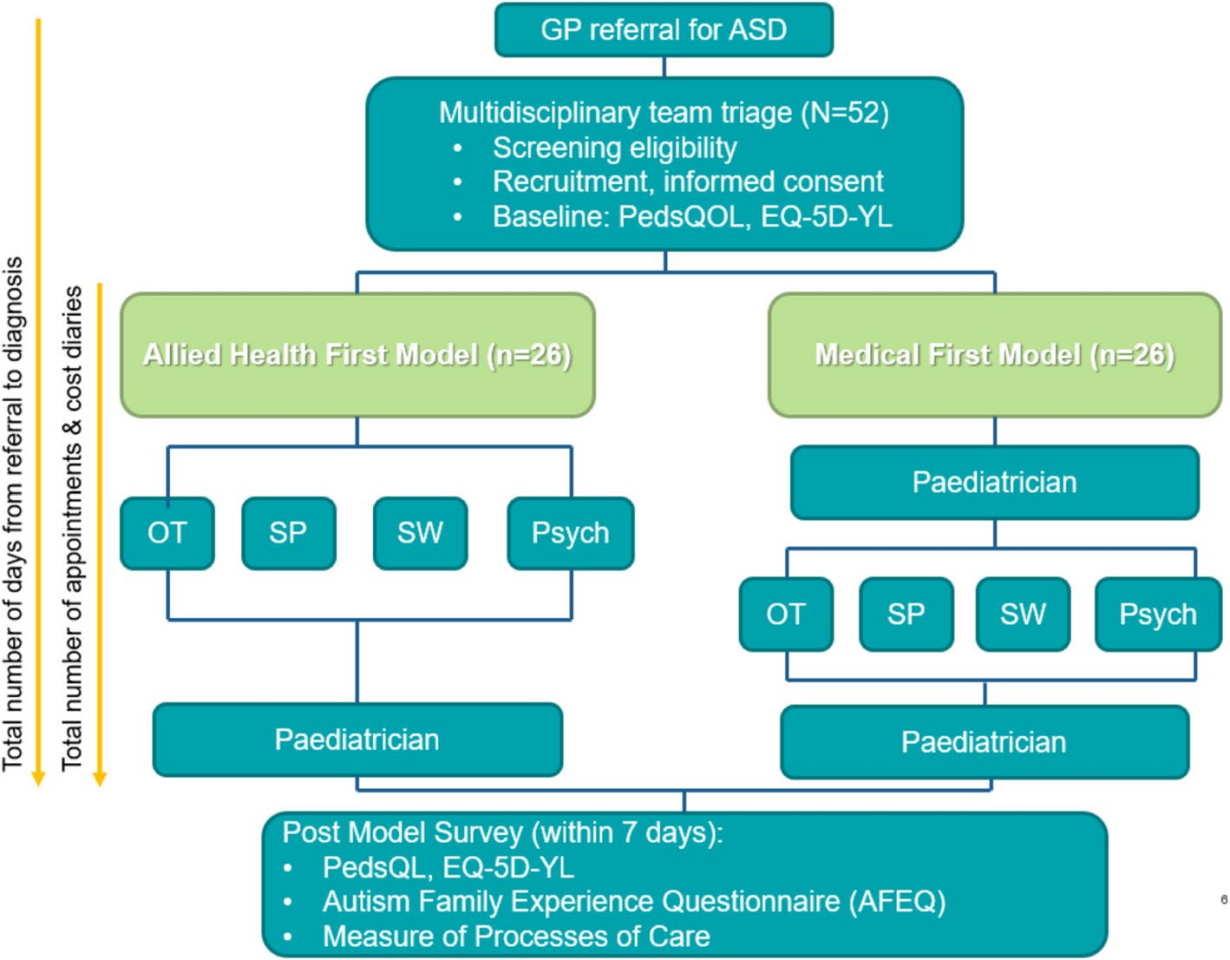
OT = Occupational Therapist; SP = Speech Pathologist; SW = Social Worker; Psych = Psychologist; PedsQL = Pediatric Quality of Life; GP = General Practitioner; ASD = Autism Spectrum Disorder; EQ-5D-Y: EuroQOL Five Dimension Youth Version

Figure 1 provides an outline of the RCT. At enrollment, the research assistant will collect baseline demographics data and complete the Peds-QL and EQ-5D-YL with the child and/or caregiver via a face to face meeting or phone review, whichever method is preferred by the caregiver. As the EQ-5D-Y questionnaire is designed for completion by children aged 8–15, we will attempt to obtain both child and parent-proxy completed measures for children in this age bracket.

At the first allied health or medical specialist appointment, the research assistant or clinician will meet the caregiver in the waiting area and provide them with a cost diary to complete. Cost diaries will be used to record all travel-related costs as well as the number of hours of caregiver time off work required to attend appointments. For subsequent allied health or medical specialist appointments, the research assistant will obtain cost diaries from the caregiver either on the day or up to 48 h post their child’s appointment at the hospital. Collection of completed cost diaries will be from any of the following modalities, whichever is preferred by the caregiver: face to face, phone review, or email.

The Research Assistant will also complete the Peds-QL, EQ-5D-YL, The Autism Family Experience Questionnaire (AFEQ) and Measure of Processes of Care with the child and/or caregiver either on the day or up to 7 days post their child’s last medical specialist appointment at the hospital. Collection of surveys will be either face to face or in a phone review, whichever is preferred by the caregiver.

For phone reviews, a reminder text message will be sent prior to the scheduled review to remind the caregiver of the scheduled date and time. A reminder text message is planned for this trial to allow caregivers the opportunity to reschedule, if a more appropriate time/date is desired. Additionally, a reminder text message is anticipated to assist with retention of participants over the trial period. Similar to previous trials conducted by the research team, a total number of three communication (e.g. text, phone, letter) attempts is allowed at any review time point [20].

### Study completion

Once all participants have reached the end of the trial period, the study investigators will request a retrospective data extraction of allied health and specialist outpatient appointment dates and costs, as well as any emergency hospital presentations and hospital admissions dates and costs, for all participants. These data are routinely collected within heath service databases.

### Safety considerations/patient safety

The research team will adhere to the principles of the Declaration of Helsinki [21] and Good Clinical Practice when conducting this study [21]. Only the study investigators affiliated with Queensland Health will have access to identifiable information, as per our ethically approved study protocol. De-identified information will only be seen by investigators on the study. Identifiable study information will be securely stored on a database within Queensland Health computer systems. All original clinical record forms will be in a locked cabinet at Caboolture Hospital, in accordance with National Health and Medical Research Council (NHMRC) guidelines and Australian regulations (e.g. minimum 15 years after the child reaches the age of 18).

### Sample size and statistical power

Accepting an alpha risk of < 0.05, a beta risk of < 0.2 and assuming a 10% risk of dropout during follow-up, *n* = 26 patients in each group is required to identify a statistically significant reduction of at least one medical appointment between groups. The sample size determination was not reliant on the standard deviation as a sample size formula input due to the number of medical appointments being considered a count variable [22].

### Statistical analyses

To account for clustering at the hospital level and repeated measures over time, multivariable mixed-effects regression models will be built to estimate the interventional effect of health related quality of life and patient experiences between and within groups. Multivariable mixed-effects regression models were chosen to to minimise bias due to group imbalance and its effects on the internal validity of the study [23]. For each model building process, variables found to be significantly associated with the outcome measure in the univariable analysis at the 10% level will be retained in the final model. As the primary predictor of interest, intervention effect will be forced into the multivariable model. Continuous outcome measures will be analysed using linear regression models. For dichotomous measures, the analysis will be by binary logistic regression, while multinomial logistic regression will be used to analyse nominal measures. Model diagnostics will be conducted on all models to ascertain their appropriateness. Each model will be tested for violations of model assumptions, the existence of influential points and goodness of fit. Tests for all outcomes will be two-tailed, with levels of statistical significance set at 5%. To assess the missingness mechanism, Little’s test for missing completely at random (MCAR) will be used.

An economic evaluation will take the form of a cost-consequence analysis, where descriptive statistics including means, standard deviations and 95% confidence intervals will be used to summarise differences across all relevant cost and outcome measures between the two models of care. This type of economic evaluation design was chosen to reflect the nature of the intervention where improvements are expected to be seen across a range of health service and patient outcomes (including family out of pocket costs, health system costs, quality of life, patient experience, patient satisfaction, wait times). The cost-consequence analysis framework allows for any changes in these outcomes to be reported clearly and transparently without the restriction of needing to identify a single outcome measure of interest, as is the case with cost-effectiveness analysis. The reader or decision maker is then able to form an opinion about the value of this service change taking account of all relevant costs and outcomes. Specific cost categories to be reported on in the analysis will include allied health and specialist outpatient appointment costs, emergency department presentations, hospital admissions costs and costs of patient (family) travel and time off work to attend appointments. EQ-5D-Y responses will be converted to a utility score using an appropriate published value set. Utility scores will be reported separately for child self-completion and caregiver proxy completion questionnaires. These utility scores will then be used to generate estimates of Quality Adjusted Life Years (QALYs) for each treatment group.

As this study is registered on the ANZCTR and occurring within Metro North Hospital and Health Service, the trial may be randomly audited by an independent study monitor at any time point in the study. Given that this study is unblinded and not comparing rates of mortality or major morbidity, a data monitoring committee will not be set up for the purposes of this specific study.

### Ethics approval

The Children’s Health Queensland Human Research and Ethics Committee, Queensland, Australia (HREC/21/QCHQ/77040) approved this trial. Enrolled children and their caregivers will be able to withdraw from the study at any time without explanation or penalty from the research team and staff at Caboolture Hospital and The Prince Charles Hospital. Written informed consent will be obtained from a parent or guardian for participants under 16 years old. Any changes to the study protocol will require additional approval from Children’s Health Queensland Human Research and Ethics Committee, Queensland, Australia. In addition, any major changes to the study protocol will also be updated on the ANZCTR accordingly.

### Trial status

This study is planned to commence recruitment in November 2021 and is planned to continue for an estimated period of 12 months. We plan to publish the trial results in relevant peer-review journals at the conclusion of the trial.

## Discussion

This randomised controlled trial will investigate the impacts of an allied health first contact model for the assessment of paediatric ASD on health service use and costs, family out of pocket costs, patient quality of life, patient satisfaction with care and patient waiting times. It is anticipated that the allied health first contact model will result in the reduction of at least one paediatrician appointment on average per child without compromising on patient quality of life and satisfaction, when compared to the traditional medical specialist first contact model.

To our knowledge, this will be the first randomised controlled trial of an allied health first contact model for the assessment of children with ASD internationally. This is a rigorous study design that will minimise the likelihood of bias in our findings. Nonetheless, some limitations exist. The type of families that consent to be involved in a research study may differ from families that choose not to be involved; this may introduce a selection bias that could limit the generalisability of our conclusions. Data collected from families on their out of pocket costs will be obtained at key time points throughout the trial, but may be subject to the effects of recall bias when asking caregivers to record costs occurring in the past. Data collected on health service use and costs, the primary outcome, will be obtained from administrative databases and are therefore expected to be comprehensive and accurate. However, this will be limited to emergency department presentations, hospital admissions, as well as allied health and specialist appointments that are provided by the health service. Data on primary or community-based care and pharmaceutical use will not be captured within this study.

The methodological and practical challenges of measuring and valuing quality of life in paediatric cohorts are well recognised, [24–26] and the evidence base continues to evolve in this field [27]. Additional challenges are present when children have mental disorders that may impact on cognition, literacy or other factors relating to their ability to respond to a questionnaire. Arguments have been made for the merits and limitations of both child-reported and parent proxy-reported completion. In this study, we will adhere to current guidance suggesting that both types should be collected simultaneously where possible [28]. Nevertheless, future studies could consider the incorporation of clinical outcomes (e.g. cognition, speech and language) in conjunction with caregiver-reported outcomes to strengthen the rationale for healthcare professionals to adopt specific service models in the assessment of ASD in children.

The findings from this study are expected to contribute to the evidence base around the costs and consequences of allied health first contact models for the assessment of children with ASD in the Australian setting. If the study hypotheses prove to be correct, this model may free up scarce health care resources while maintaining, or potentially improving, patient and family quality of life and experience of care. These findings may be useful in informing the wider adoption of these models in Australia and internationally, particularly in healthcare settings where medical specialist shortages exist.

## Data Availability

Data sharing is not applicable to this article as no datasets were generated or analysed during the current study.

## Abbreviations

AFEQ: The Autism Family Experience Questionnaire
ANZCTR: Australia and New Zealand Clinical Trials Registry
ASD: Autism Spectrum Disorder
HREC: Human Research Ethics Committee
NHMRC: National Health and Medical Research Council
PedsQL: Pediatric Quality of Life Inventory (PedsQL)
QoL: Quality of life
SEIFA: Socio Economic Index For Area
EQ-5D-Y: EuroQOL Five Dimension Youth Version
